# Leveraging EUnetHTA’s conceptual framework to compare HTA decision drivers in France, Italy, and Germany from a manufacturer’s point of view

**DOI:** 10.1186/s13561-018-0201-y

**Published:** 2018-09-21

**Authors:** Giovanni Giuliani, Frederic Chassagnol, David Traub, Marlene Gyldmark, Ansgar Hebborn, Pierre Ducournau, Jörg Ruof

**Affiliations:** 1 Roche S.p.A, Monza, Italy; 2 Roche SAS, Boulogne-Billancourt Cedex, France; 3 Roche Pharma AG, Grenzach-Wyhlen, Germany; 4 F. Hoffmann La Roche, Basel, Switzerland; 5 Medical School of Hanover, Hanover, Germany; 6 r-connect ltd, Hauensteinstr. 132, 4059 Basel, Switzerland

**Keywords:** Appraisal, EUnetHTA Core model, Health technology assessment, Innovation, HAS, AIFA, G-BA

## Abstract

**Background:**

Health Technology Assessments (HTA) procedures differ substantially across the various European countries. We reviewed recent appraisals of a pharmaceutical manufacturer in three major European markets (France; Italy; Germany) and identified and categorized related decision drivers.

**Methods:**

New marketing authorisation between January 2011 and August 2017, and Roche being the Marketing Authorization Holder, were included. Outcome of HTA appraisals by the Haute Autorité de Santé (HAS), Agenzia Italiana del Farmaco (AIFA), and Federal Joint Committee (Gemeinsamer Bundesausschuss, G-BA) were reviewed. Respective decision drivers were identified and commonalities and differences across the three countries were determined leveraging the EUnetHTA conceptual taxonomy (i.e. the 9 domains of the EUnetHTA core model).

**Results:**

Within that time period Roche received European marketing authorization for eight new molecular entities (10 indications, respectively). Outcome of HTA appraisals was heterogeneous across the three countries. However, the four clinical domains of the EUnetHTA core model were driving the national HTA appraisals, with the clinical effectiveness domain being of most importance. Important drivers related to the other three clinical domains included the target patient population (subgroups, Germany), the current management of the condition (unmet need, Italy), the regulatory status (Orphan Designation, Germany), as well as safety considerations (all three countries). Average time between EMA approval and full commercial availability of new medicines was 63 (Germany), 459 (Italy), and 557 days (France).

**Conclusions:**

The clinical domains of the EUnetHTA framework are mainly driven by national HTA appraisals, providing a suitable starting point for further developing a joint European view on value and evidence. Underlying topics and issues still reveal considerable differences.

## Background

Over the past decades Health Technology Assessments (HTAs) for innovative medicines have become a standard feature in almost all European countries. The ‘Agenzia Italiana del Farmaco’ (AIFA) in Italy was established in 2003 [1]. In 2004 the French law was published introducing the ‘Haute Autorité de Santé’ (HAS, [2]), and since 2011 the German Social Code Book V requires the Federal Joint Committee (Gemeinsamer Bundesausschuss, G-BA) to perform a comprehensive benefit assessment of pharmaceuticals [3].

While all those institutions aim for an optimization of the health benefits for their respective population the applied Health Technology Assessment (HTA) procedures and the appraisals largely differ across the various European countries. Most obviously some northern European countries and the United Kingdom heavily rely on a direct comparison of costs and outcomes by means of a cost-effectiveness analysis while e.g. in France and Germany, the determination of the additional clinical benefit versus the current standard of treatment is conceptually separated from the subsequent determination of medication costs and other economic consequences.

Those heterogeneities of HTA appraisals across Europe are well known and more recently the European Commission has made a regulatory proposal to strengthening the cooperation between the EU member states on Health Technology Assessments [4]. Conceptually, the European network for Health Technology Assessment (EUnetHTA) has been taking a leading role in the effort to develop a joint view on evidence generated within clinical trial programmes for medicines [5, 6]. About a decade ago, EUnetHTA developed the HTA Core Model^®^, a science-based framework for a joint assessment of core dimensions of value, which in the meantime has undergone several substantial revisions and improvements [7]. Based on the conceptual framework of the HTA Core Model^®^, EUnetHTA offers the conduct of rapid joint assessments of the relative clinical effectiveness of medicines based on the voluntary submission of a pharmaceutical manufacturer. Within those ‘Joint Assessments’ two or more country HTA agencies work together to author the respective reports, other HTA agencies act as reviewers of the joint work [8].

From a pharmaceutical manufacturer’s point of view, better alignment of the heterogeneous national HTA approaches with regard to clinical HTA domains (i.e. an aligned set of required methodological criteria) is desirable with regards to the feasibility and economic viability of medicine development programmes in a global and highly competitive environment. We therefore analysed all Roche pharmaceutical appraisals between 2011 and 2017 across three major European markets (France, F; Italy, I; Germany, G). Those markets were selected due to the size of their economic impact across Europe. Key decision drivers per product were identified in each of the countries and the EUnetHTA core model terminology was leveraged to identify the most decisive value domains, topics, and issues.

## Methods

New molecular entities and their line extensions receiving marketing authorisation between January 2011 and August 2017, and Roche being the Marketing Authorization Holder, were identified and included in this analysis. The timeline of January 2011 was chosen as in 2011 Germany, as last major European market, introduced a systematic HTA process as integral part of the national pricing and reimbursement process (AMNOG).

Three analysis steps were conducted:

i) review of national HTA appraisal outcomes in the 3 European countries;
ii) qualitative review and categorization of key appraisal decision drivers;
iii) identification of commonalities and differences across countries

### Step 1 – Outcomes of HTA appraisals

Time of marketing authorization approval by the European Medicines Agency (EMA) was derived from the EMA homepage (www.ema.europa.eu). Timing and outcomes of HTA appraisals by HAS [9], AIFA [10], and G-BA [11] were analyzed using public sources:

- France: The Actual Clinical Benefit (SMR: Service médical rendu; range from substantial, moderate, low, to insufficient), Clinical Added Value (ASMR: Amélioration du service medical rendu; range from I: major, II: substantial, III: moderate, IV: minor, to V: no clinical value added) as well as the timing of HAS decisions were derived from the HAS official website (www.has-sante.fr) and from the website of the Ministry of Health and the Ministry of Budget [12]. Commercialisation in France is effective after HAS opinion and negotiation of the price with the economics committee.
- Italy: The product class (A: essential, fully reimbursed; H: only fully reimbursed in hospitals; C: not reimbursed) as well as specific conditions for reimbursement were obtained from the official journal of AIFA (www.gazzettaufficiale.it). Local marketing authorisation and commercialisation takes place at the same time as AIFA’s decision is officially published. Respective dates were also derived from the ‘Gazzetta Ufficiale’.
- Germany: Appraisals were reviewed on the G-BA’s homepage (www.g-ba.de). Information on the additional benefit (major, important, minor, non-quantifiable, no additional benefit, less additional benefit), the evidence rating (proof, indication, hint), and subgroups were extracted. Commercialisation in Germany occurs after EMA approval and requires a listing of the initial price (prior to negotiation) in the ‘Lauer-Taxe’, the official price list of medicines [13].

### Step 2 – Qualitative review and categorization of key appraisal decision drivers

National appraisals were qualitatively reviewed. Key decision topics supporting or limiting positive appraisals per medicine and country were identified by one author (JR) and reviewed by the respective country affiliate author (France: FC; Italy: GG; Germany: DT). Key decision drivers were mainly derived from the section ‘Conclusions de la Commission’ (F; HAS appraisal), the ‘Gazzetta Ufficiale’ (I; AIFA appraisal), and the ‘Gesamtbetrachtung’ (G, G-BA appraisal, ‘Tragende Gründe’), but all available published information as well as process experience of the respective country affiliate authors throughout the process was reflected in this qualitative analysis.

The decision drivers, that originate from the national HTA approaches in F, I, G were categorized according to the EUnetHTA core model domains. The EUnetHTA core model consists of three structural levels: a total of 9 ***domains*** (i: health problem and current use of technology (CUR); ii: description and technical characteristics of technology (TEC); iii: safety (SAF); iv: clinical effectiveness (EFF); v: cost and economic evaluation (ECO); vi: ethical analysis (ETH); vii: organisational aspects (ORG); viii: patient and social aspects (SOC); ix: legal aspects (LEG)) as well as the related ***topics*** and ***issues***, with the issue representing the most granular level [14]. Country affiliate authors were asked to link the identified key discussion points of their national appraisal to the topics and issues of the EUnetHTA core model. In order to minimize a possible bias in the categorization due to the subjective views of one author, all other authors conducted the same exercise. Results were compared, and discrepancies discussed among all authors.

### Step 3 – Identification of commonalities and differences across countries

Based on the identification and categorization of national decision drivers (Step 2), specific decision patterns per country were identified and commonalities and differences across the three countries were determined.

While all the preparatory work was done by two authors (GG, JR), the second part of step two (categorization of national decision drivers) as well as step 3 (qualitative discussion around commonalities and differences in decision drivers across the three countries) were conducted during a one - day workshop with all authors. Within that workshop, final agreement was reached i) regarding national decision drivers per country; ii) grouping of decision drivers according to the EUnetHTA core model domains; and iii) commonalities and differences in decision drivers across the three countries.

## Results

### Included medicines

Between January 2011 and August 2017 Roche pharmaceuticals received European marketing authorization for eight new molecular entities (10 indications). All except one (Pirfenidon, Idiopathic Pulmonary Fibrosis) covered oncological conditions. Pirfenidon received EMA approval in February 2011. The initial process was managed by InterMune which was acquired by Roche Pharmaceuticals in August 2014. Venetoclax (Chronic Lymphocytic Leukaemia) was not included as AbbVie is the Marketing Authorisation Holder across Europe. Average time between EMA approval and full commercial availability of new medicines was 63 (Germany), 459 (Italy), and 557 days (France).

### Step 1 –outcome of HTA appraisals

The Overview of HTA appraisals in F, I, and G are displayed in Table 1.

- France: The time gap between EMA approval and HAS appraisal ranged between 112 days (Cobimetinib) and 380 days (Pirfenidon) with a mean of 227 days. SMR ratings were substantial for Vemurafenib, Pertuzumab (metastatic), Vismodegib, Trastuzumab Emtansine, Obinutuzumab in both indications, Cobimetinib, and Alectinib, moderate for Pirfenidon (2nd appraisal), and insufficient for Pertuzumab (neoadjuvant). ASMR ratings were substantial for Trastuzumab Emtansine, moderate for Vemurafenib, Pertuzumab (metastatic), Obinutuzumab (CLL), and Cobimetinib, minor for Pirfenidon, Vismodegib, and Alectinib, and no added benefit for Obinutuzumab (FL).
- Italy: The official publication in the Gazzetta Ufficiale occurred between 164 days (Obinutuzumab CLL) and 837 days (Vismodegib) after EMA approval (mean 465 days). Assigned class was H for all of the products. Obinutuzumab had a class C in the first CLL appraisal which was revised in February 2017. Pertuzumab in its first indication (metastatic) achieved an ‘innovation designation’ while no reimbursement was obtained in the neoadjuvant setting. Trastuzumab Emtansine received a ‘potential innovation’ designation. Payment by result schemes were applied to Vemurafenib, Trastuzumab Emtansine, and Cobimetinib. Cost sharing procedures were implemented for Pertuzumab, Vismodegib and Obinutuzumab (CLL). Alectinib has not yet been fully negotiated.
- Germany: Appraisals generally occurred on average 207 days after EMA approval, with Pirfenidon (appraisal 381 days after EMA approval) being the exception (which was due to the transition period after AMNOG came to effect). Additional subgroups beyond EMA indications were assigned to Pertuzumab (metastatic), Vismodegib, Trastuzumab Emtansine, and Alectinib. An additional benefit was assigned to Vemurafenib, Pirfenidon (Orphan Designation), Pertuzumab (metastatic patients with visceral metastases only), Vismodegib (locally advanced patients only), Trastuzumab Emtansine (patients previously treated with Anthracyclines), Obinutuzumab (Orphan Designation in both indications: CLL and FL), Cobimetinib, and Alectinib (patients eligible for chemotherapy). No additional benefit was assigned to Pertuzumab (neoadjuvant) and the remaining subgroups of Pertuzumab metastatic, Vismodegib, Trastuzumab Emtansine, and Alectinib.

**Table 1:** Timing and Outcome of HTA appraisals in France, Italy, and Germany since 2011

Medicine	EMA Appro-val	Indication	France	Italy	Germany
Vemurafenib	Feb 17th 2012	Melanoma	1st Appraisal: Oct 3rd^,^ 2012 2nd Appraisal: Mar 22nd, 2017 Publication Price: Feb 1st, 2013 Both Appraisals: SMR: substantial ASMR: moderate (level III)	Publication: Jun 4th, 2013; Class H Payment by Result scheme and sales cap	1st appraisal: Sep 6th, 2012 2nd appraisal: Mar 6th, 2014 Both appraisals: Indication considerable benefit
Pirfenidon (Orphan Designation)	Feb 28th, 2011	Idiopathic Pulmonary Fibrosis	1st Appraisal: Mar 14th, 2012 2nd Appraisal: Feb 18th^,^ 2015 Publication Price: Oct 16th, 2012 SMR 1st Appraisal: Low SMR 2nd Appraisal: Moderate ASMR: minor (level IV)	1st Publication: Jun 14th, 2013 Class H ‘success fee’ agreement and stopping rule 2nd Publication: Jul 3st, 2015 Removal of ‘success fee’ and stopping rule due to new evidence submitted	Appraisal: Mar 15th, 2012 Additional benefit non-quantifiable
Pertuzumab 1st Indication Metastatic	Mar 4th 2013	Her2+ metastatic Breast Cancer	Appraisal: Jul 24th, 2013 Publication Price: Dec 13th, 2013 SMR: substantial ASMR: moderate (level III)	Publication: June 23rd, 2014 Class H ‘Innovation designation’	Appraisal: Oct 1st, 2013 • Visceral Metastases: Hint Considerable benefit • Non-visceral Metastases: no additional benefit • Locally recurrent: no additional benefit
Pertuzumab 2nd Indication Neoadjuvant	Jul 28th 2015	Neo-adjuvant	Appraisal: Jul 6th, 2016 SMR: insufficient ASMR: not applicable	Publication: Jan 5th, 2017 No reimbursement	Appraisal: Feb 18th, 2016 No additional benefit
Vismodegib	Jul 12th 2013	Locally advanced or metastatic Basal Cell Carcinoma	Appraisal: Dec 18th, 2013 Publication Price: Sept 2nd, 2015 SMR: substantial ASMR: minor (level IV)	Publication: April 9th, 2015 Class H Cost sharing agreement	1st appraisal: Feb 6th, 2014 2nd appraisal: Aug 4th, 2016 Both appraisal: • Metastatic BCC: no additional benefit • Locally Advanced BCC: Hint Minor Benefit
Trastuzumab Emtansine	Nov 15th, 2013	Her2+ Breast Cancer	Appraisal: Mar 19th, 2014 Publication Price: Nov 14th, 2014 SMR: substantial ASMR: substantial (level II)	Publication: Sep 26th, 2014 Class H ‘Potential Innovation’ designation Payment by Result scheme	Appraisal: Jun 19th, 2014 • Locally Advanced: no additional benefit • Previous therapy includes Anthracycline: Indication considerable benefit • Previous therapy without Anthracycline: no additional benefit
Obinutu-zumab 1st indication (Orphan Designation)	Jul 23rd 2014	Chronic Lymphocytic Leukaemia	Appraisal: Feb 18th, 2015 Publication Price: Dec 24th, 2015 SMR: substantial ASMR: moderate (level III)	1st Publication: Jan 3rd, 2015 Class C 2nd Publication: Feb 24, 2017 Class H Reimbursed with cost sharing scheme	Appraisal: Feb 5th, 2015 Non-quantifiable additional benefit
Obinutu-zumab 2nd indication (Orphan Designation)	Jun 13th, 2016	Follicular Lymphoma	Appraisal: Mar 8th, 2017 SMR: substantial ASMR: no (level V)	Publication: Aug 31st, 2017 Class H Reimbursed with discount (removal of previous cost sharing agreement)	Appraisal: Dec 16th, 2016 Non-quantifiable additional benefit
Cobimetinib	Nov 20th, 2015	Melanoma	Appraisal: Mar 16th, 2016 Publication Price: Feb 16th; 2017 SMR: substantial ASMR: moderate (level III)	Publication: Oct 1st, 2016 Class H Reimbursed with Payment by Result Scheme	Appraisal: Jun 2nd, 2016 Indication Considerable benefit
Alectinib	Feb 16th 2017	Non-small lung cancer	Appraisal: Dec 13th, 2017 Publication Price: Pending SMR: substantial ASMR: minor (level IV)	Publication: Final Publication pending	Appraisal: Oct 19th, 2017 • Patients eligible for DCP: Hint Minor benefit • Patients not eligible for DCP: no additional benefit

*Abbreviations: ASMR* Amélioration du Service Médical Rendu, *CDF* Cancer Drug Fund, *DCP* Docetaxel, or Pemetrexed, or Ceritinib, *ERG* Evidence Review Group, *FVC* Forced Vital Capacity, *ICER* Incremental Cost Effectiveness Ratio, *QALY* Quality Adjusted Life Year, *SMR* Service Médical Rendu

* 2nd opinion was conducted as Budget Impact by Pirfenidon sales was considered significant

**Fig. 1:**
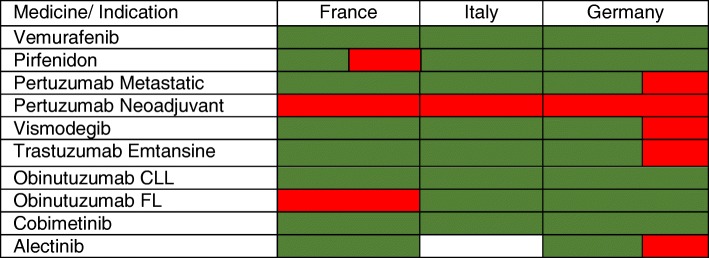
HTA recommendations across France, Italy, and Germany. Green: ASMR ≤ 4 (France); Class H or A categorization (Italy); Additional benefit (major, considerable, minor, or non-quantifiable) (Germany). Red: any other appraisal. White: appraisal not publicly available yet

Figure 1 illustrates the level of alignment across the three countries on a binary level (positive/negative appraisal): while appraisals overall were aligned in 4 indications (positive appraisals in F, I, G for Vemurafenib, Obinutuzumab CLL, and Cobimetinib; negative appraisal in F, I, G, for Pertuzumab Neoadjuvant), partial alignment occurred in 5 indications (Pirfenidon, Pertuzumab Metastatic, Vismodegib, Trastuzumab Emtansine, Alectinib) and no alignment occurred in Obinutuzumab FL which received an ASMR V in France and a positive appraisal in Italy and Germany.

### Step 2 – Qualitative review and categorization of key decision drivers

Tables 2 and 3 provide an overview of key decision drivers within each of the 10 appraisals.

**Table 2:** Key appraisal decision drivers, categorized according to the EUnetHTA core model terminology

Medicine	Appraisal France	Appraisal Italy	Appraisal Germany
Pirfenidon	1) EFF (moderate effect on FVC; Mortality impact unclear) 2) CUR (limited to patients with FVC ≥ 50% and DLCO ≥ 30%) 3) SAF (Tolerability Monitoring) 4) ORG (smoking cessation required)	1) EFF (initial appraisal: limited and inconsistent data; second appraisal: new clinical data) 2) CUR (lack of treatment alternatives) 3) ECO (treatment costs/ budget impact) 4) SAF (initial safety concerns)	1) TEC (Orphan Designation) 2) EFF (Patient relevance of FVC was challenged; trial outcomes considered not consistent) 3) CUR (Stage of Disease difficult to determine)
Vemurafenib	1) EFF (OS & PFS benefit) 2) SAF (concerns regarding 2nd skin cancer) 3) CUR (Targeted therapy)	1) EFF (Clinical Data; OS and PFS benefit) 2) CUR (high unmet need) 3) ECO (concerns budget impact) 4) TEC (Novelty of Treatment)	1) EFF (OS benefit considered relevant No additional benefit in morbidity or Quality of Life. PFS not accepted) 2) CUR (Severity of Condition) 3) SAF (Side effects considered manageable)
Pertuzumab 1st indication Metastatic	1) EFF (Treatment expected to have substantial impact on morbidity and mortality; median OS not reached and no QoL benefit has been shown)	1) EFF (OS benefit) 2) TEC (High clinical value recognized through Innovation designation) 3) CUR (severity of condition) 4) ECO (budget impact concerns)	1) EFF (Additional benefit only in patients with visceral metastasis driven by OS benefit. No Morbidity of QoL benefit accepted. PFS considered not relevant to patients) 2) CUR (G-BA separated 3 patient groups; no additional benefit in patients with non-visceral and locally advance disease) 3) SAF (Safety results difficult to interpret as based on different observation periods)
Pertuzumab 2nd indication Neoadjuvant	1) EFF (Clinical Data insufficient; based on proof-of-concept study only)	1) EFF (Proof of concept study only; Surrogate endpoint was challenged)	1) EFF (Validity of surrogate endpoint pCR considered unclear; Trial did not show differences in OS and relapse rates)
Vismodegib	1) CUR (absence of valid treatment alternative) 2) EFF (efficacy demonstration limited to one none comparative trial) 3) SAF (high efficacy/ adverse effects ratio)	1) CUR (High Unmet medical need) 2) TEC (Innovative Technology recognized) 3) EFF (proof-of-concept trial with single arm design is considered premature) 4) SAF (safety concerns)	1) EFF (Single Arm trial controversially discussed; Externally visible lesions implicitly accepted as relevant to patients) 2) CUR (Discussion about spontaneous remissions)
Trastuzumab Emtansine	1) EFF (PFS & OS advantage) 2) SAF (acceptable safety profile)	1) EFF (OS benefit) 2) TEC (Clinical value recognized through Innovation designation) 3) CUR (Severity of condition acknowledged) 4) ECO (Economic concerns regarding budget impact) 5) SAF (No additional safety signals)	1) EFF (Additional benefit in patients with prior Anthracycline treatment based on OS benefit; QoL benefit acknowledged) 2) CUR (G-BA separated 3 patient groups and requests Anthracycline as comparative treatment in subset of Her2+ patients) 3) SAF (reduction in side effects e.g. diarrhoea)
Obinutuzumab CLL	1) EFF (Improvement in PFS and Minimal Residual Disease but no OS demonstrated) 2) SAF (Toxicity of dual therapy containing Obinutuzumab greater than with Rituximab)	1) CUR (Therapeutic alternative available) 2) EFF (Lack of OS benefit was critically reviewed)	1) TEC (Additional benefit guaranteed due to Orphan Designation) 2) EFF (OS data considered immature; PFS not considered relevant to patients; No QoL benefit) 3) SAF (Adverse events rate with Obinutuzumab higher than with Rituximab)
Obinutuzumab FL	1) EFF (Improvement in PFS but no OS demonstrated; many issues regarding clinical trial design were raised)	1) EFF (No OS benefit) 2) CUR (unmet need recognized)	1) TEC (Additional benefit guaranteed due to Orphan Designation) 2) EFF (OS effect based on low number of events; VAS of EQ-5D with positive trend)
Cobimetinib	1) EFF (Improvement in PFS and OS) 2) CUR (Recommended as first line treatment option equal to trametinib/dabrafenib)	1) EFF (Clinical value recognized; OS/PFS) 2) TEC (Innovative Technology recognized)	1) EFF (Moderate OS benefit; positive QoL effects; mostly positive impact on disease symptoms (pain, sleep, fatigue).
Alectinib	1) EFF (Improvement in PFS; partial responses on cerebral metastases) 2) SAF (Hepatic and gastrointestinal side effect)	1) EFF (Clinical value recognized; PFS) 2) CUR (Unmet need recognized)	1) CUR (Separation of subgroups: Patients eligible for DCP yes/ no) 2) SAF (Less side effects vs DCP) 3) EFF (no OS benefit; cross over rate acknowledged; PFS and CNS Response rates not considered relevant for patients)

EUnetHTA Core Model Domains: *CUR* Health Problem and Current Use of Technology, *TEC* Description and technical characteristics of technology, *SAF* Safety, *EFF* Clinical Effectiveness, *ECO* Cost and economic evaluation, *ORG* organisational aspects, *SOC* Patients and Social Aspects, *LEG* Legal Aspects

*Other Abbreviations: CDF* Cancer Drug Fund, *CNS* Central nervous system, *CLL* chronic lymphocytic leukaemia, *DCP* Docetaxel, or Pemetrexed, or Ceritinib, *DL*_*CO*_ Diffusing Capacity for Carbon Monoxide, *EQ-5D* EuroQoL 5D questionnaire, *ERG* Evidence Review Group, *FL* Follicular Lymphoma, *FVC* Forced Vital Capacity, *PbR* Payment by Result, *pCR* pathologic complete response, *HAS* Haute Autorité de Santé, *ICER* Incremental Cost Effectiveness Ratio, *IPF* Idiopathic Pulmonary Fibrosis, *NICE* National Institute for Health and Care Excellence, *OS* Overall Survival, *PFS* Progression free survival, *QoL* Quality of Life, *QALY* Quality Adjusted Life Year, *VAS* Visual Analogue Scale

**Table 3:** Number of decision drivers related to the respective EUnetHTA core model domain: review of 10 products across France/ Italy/ Germany

Country	CUR	TEC	SAF	EFF	ECO	ETH	ORG	SOC	LEG
France	3	1	6	10			1		
Italy	8	5	3	10	4				
Germany	6	3	4	10					

EUnetHTA Core Model Domains: *CUR* Health Problem and Current Use of Technology, *TEC* Description and technical characteristics of technology, *SAF* Safety, *EFF* Clinical Effectiveness, *ECO* Cost and economic evaluation, *ETH* Ethical analysis, *ORG* organisational aspects, *SOC* Patients and Social Aspects, *LEG* Legal Aspects

Across all three countries, the most relevant decision drivers were derived from the 4th domain of the EUnetHTA core model; *Clinical Effectiveness (EFF).* Key clinical endpoints such as Overall survival were positively driving HTA appraisals for Vemurafenib, Pertuzumab (1st indication; metastatic patients, showing a positive OS trend which was confirmed in later analyses), Trastuzumab Emtansine, and Cobimetinib. Acceptance of endpoints other than Overall survival differed across the three countries. Despite different views on Progression Free Survival (PFS) within G-BA, this endpoint is considered a surrogate endpoint and as such not relevant to patients. Differently both, improvement in PFS as well as MRD (Minimal Residual Disease) was taken into account by HAS in their appraisal of Obinutuzumab’s first indication. Concerns with the specific features of underlying study designs where raised by all three authorities, HAS, AIFA, and G-BA after reviewing the clinical trial programs of Vismodegib and Pertuzumab in the neoadjuvant setting. Further differences across the countries were identified with regards to appropriate comparative therapy. E.g. France considered Bendamustine Monotherapy not in line with French clinical practice in the appraisal of Obinutuzumab in Follicular Lymphoma and, on the other side, acknowledged that there are no suitable comparative therapies for Pertuzumab in metastatic Breast Cancer and for Vismodegib in advanced Basal Cell Carcinoma. Instead G-BA did not assign a comparative therapy for Obinutuzumab in Follicular Lymphoma as Obinutuzumab has an Orphan designation. Comparative therapies for Pertuzumab in metastatic Breast Cancer and Vismodegib were differentiated according to assigned subgroups. As we considered those concerns covered by the topic ‘Test Accuracy’, we categorized them as ‘Clinical Effectiveness’ issue within the EUnetHTA terminology.

In Italy, eight decision drivers (France 3, Germany 6, respectively) were derived from the domain ‘*Health Problem and Current Use of Technology’, CUR*. They were mostly related to recognition of ‘High Unmet Medical Need’ and ‘Lack of Alternative Treatment Alternatives’ (Topic ‘Target Condition’ within the EUnetHTA terminology). Also, in Germany the EUnetHTA domain CUR contained important decision drivers. However, a characteristic feature of the German system is that the ‘Target Population’ (which belongs to the EUnetHTA domain: ‘CUR’) is frequently broken down in various subgroups with different benefit levels being assigned to each of those subgroups. Within the reviewed appraisals, the G-BA applied subgroups to Pertuzumab, Vismodegib, Trastuzumab Emtansine, and Alectinib.

The EUnetHTA domains ‘Safety’ (SAF) and ‘Description and technical characteristics of Technology’ (TEC) also contained important decision drivers. The benign safety profile of Trastuzumab Emtansine was positively acknowledged by all three health authorities. Also, in the German Alectinib appraisal the improved side effect profile versus the comparative arm with chemotherapy contributed to the positive appraisal. In Germany the TEC domain was driving decision for Obinutuzumab and Pirfenidon (Topic ‘Regulatory Status’) due to Orphan designation. As both the CUR, as well as the TEC domain include a topic on ‘Regulatory Status’, the categorization of this decision driver was based on an aligned decision of the authors.

Differently, the non-clinical EUnetHTA domains (ECO, ETH, ORG, SOC, LEG) were by far less important within the reviewed national HTA appraisals. Only in Italy the envisioned budget impact and real-world utilization was impacting some of the HTA decisions. In France, smoking cessation, and certain respiratory function criteria (forced vital capacity, FVC ≥ 50% and diffusing capacity for carbon monoxide DLCO ≥ 30%) are required prior to therapy with Pirfenidon for pulmonary fibrosis. We considered this condition related to the EUnetHTA domain ‘Organisational Aspects’ (ORG) and to the respective topic ‘Health Delivery Process’ (Item: ‘What kinds of co-operation and communication of activities have to be mobilised’).

### Step 3 – Identification of commonalities and differences across the three countries

With regards to the EUnetHTA core domains several characteristic features were identified:

- Across all three countries the clinical domains (CUR; TEC; SAF; EFF) were by far more important in driving HTA decisions than the five non – clinical domains (ECO; ETH, ORG, SOC, LEG). Only Italy included the ECO domain in some of their appraisals
- The ‘Clinical Effectiveness’ domain was most important in driving national HTA decisions. While all countries accepted ‘Overall Survival’ as an endpoint, some also recognized surrogate endpoints within their appraisals.
- Implementation of the CUR domain differed across countries, with Germany mostly focussing on the topic ‘Target Population’ (i.e. Subgroups) and Italy recognizing unmet medical need and lack of treatment alternatives.
- Also, the implementation of the TEC differed across countries, with Italy recognizing innovation designation and novelty of treatment while the three respective decisions in Germany (both indications of Obinutuzumab and Pirfenidon) were driven by the specific regulation for medicines with Orphan designation.
- The impact of Safety (SAF) was heterogeneous across the countries and no specific patterns were identified.

## Discussion

Recently, the European Commission proposed a regulation strengthening the cooperation amongst EU Member States for assessing health technology [15]. Within that proposal, rules for the conduct of clinical benefit assessments and a corresponding early scientific consultation process are suggested that are in line with the EUnetHTA framework and corresponding current EUnetHTA activities. Four EUnetHTA domains in particular (CUR; TEC; SAF; EFF) were considered suitable for joint HTA assessments at EU level. In line with the focus of this European Commission regulatory proposal our analysis revealed that primarily those four domains are used for the appraisals by HAS, AIFA, and G-BA. While AIFA frequently includes some economic components in their decisions, both France and Germany at least claim to separate the clinical appraisals from cost or wider economic considerations that may also influence price negotiations. This focus on the clinical assessment component is an important pre-requisite for the development of an aligned European view on the common trunk of clinical evidence that is coming out of each pivotal clinical trial programme. Despite differences in manufacturer submissions within the three countries the detailed description of the clinical trial data is the core component of data required for the HTA appraisals in those three countries. However, we also identified considerable differences between the three countries with regards to their HTA approach:

- Different from e.g. Germany full commercial availability of innovative medicines in France requires HAS appraisal and subsequent price agreement with the Healthcare Products Pricing Committee (CEPS). Time between EMA approval and HAS appraisal averaged at 227 days in our review and full commercialisation in France occured 557 days post EMA approval, indicating a major delay in market access for innovative products [16].
  - ◦ In France, the two components of HTA appraisal include clinical benefit (SMR) as well as added benefit (ASMR), with the latter focussing on relative effectiveness analysis. While the SMR is based on criteria such as the efficacy, safety, place in therapeutic strategy, existence of therapeutic alternatives, severity of condition, and public health impact, the ASMR determines the relative value of the medicine compared to the current standard of care. Both SMR and ASMR appreciation experienced a shift towards clinical evidence i.e. the EUnetHTA EFF domain over the past decade [17]. By far the most frequently assigned SMR category is ‘important’, while most commonly ASMR appraisals reveal a category of ‘V’ i.e. no improvement versus current standard. Within their annual activity overview 2016, HAS reported more than 750 (re-) appraisals with only 25 thereof considered a therapeutic progress (i.e. ASMR ≤ 4) versus current standard of care. Nevertheless, in only 7 of the innovative products and 20 of the re-evaluated products SMR was considered insufficient [18]. Thus, despite recent discussions about a reform of the SMR-ASMR system [17] the HTA practice including the two ratings prevailed, providing HAS with an opportunity to assign different SMR levels also to products without additional benefit. For all but two of the appraisals in our review a clinically added value was proven, with Obinutuzumab in follicular lymphoma still receiving a substantial SMR despite an ASMR ‘V’ rating.
- The clinical decision drivers within the Italian appraisals are less transparent than in France or Germany. While AIFA’s appraisal decisions are publicly available within the Gazzetta Ufficiale, the level of detail regarding HTA decision criteria within those reports is limited. Nevertheless, an advantage in Overall Survival and a high level of unmet need with a lack of treatment alternatives were identified as strong drivers of a positive AIFA decision. Importantly, the introduction of innovative pharmaceuticals in Italy is usually conducted via some kind of a ‘managed access’ scheme. Almost all of the medicines within our review had some kind of a ‘payment by result’ or ‘cost sharing scheme’. Thus, potential budget impact is proactively managed by AIFA. However, while those considerations are more explicitly discussed in Italy, and therefore more transparent, budget impact considerations are also impacting HTA appraisals in France and Germany. Half of the votes within each G-BA appraisal are derived from the ‘National Association of Statutory Health Insurance Funds’ who later on in the process is in charge of the price negotiations. This principle of G-BA’s governance indicates a strong link between HTA appraisals and price and budget impact considerations.
- Characteristic features of the German HTA appraisals and the respective decision drivers are the reflection of added benefit in relation to a specific comparator as well as the underlying level of evidence (proof/ indication/ hint/no evidence), the separation of subgroups, a stringent definition of ‘patient relevance’ which often precludes the consideration of key clinical trial endpoints, and the special regulation for Orphan Medicines where an additional benefit is granted by the law even if G-BA sometimes seems sceptical about it (e.g. Obinutuzumab FL). Furthermore, Germany applies a very strict time schedule to its HTA appraisals which is embedded in the respective law.

Despite those differences in national HTA approaches across the three countries, the focus on the clinical components of the EUnetHTA framework is an important commonality. Also, further alignment between the countries with regards to appropriate therapy seems to be achievable at least in medicines such as Vismodegib and Pertuzumab (1st indication) where e.g. France acknowledges a lack of an appropriate comparator and Germany determines ‘best supportive care’ as the appropriate comparator. This differs e.g. from the UK, were cost-effectiveness considerations (i.e. the ECO domain) are the primary drivers of NICE’s decisions, leading to major differences in respective appraisals: e.g. both Cobimetinib and Vismodegib received a negative appraisal by NICE, or Pertuzumab in the neoadjuvant indication being considered cost–effective. However, in contrast to e.g. Germany, NICE seems to have a different quantitative approach to handling uncertainty in evidence with regards to endpoints (e.g. pathologic complete response in the neoadjuvant indication of Pertuzumab) or the acceptance of indirect treatment comparisons.

An important limitation of our analysis is the limitation to three European markets only. As the EMA market authorization applies to more than 30 markets, part of the future research agenda is an expansion of the scope of this analysis beyond France, Germany, and Italy. Within our analysis we initially also considered the inclusion of the UK. However, market access of most of the oncology medicines reviewed in this analysis were managed via the Cancer Drug Fund (CDF). This heavily impacted procedures and timelines of the NICE (National Institute of Clinical Excellence) processes. Therefore, UK was excluded from the final analysis set. Nevertheless, markets with a focus on cost-effectiveness should be explored further and also inclusion of products beyond Roche’s portfolio is an important research topic for the future.

## Conclusion

In conclusion, the focus of France, Italy, and Germany on the clinical domains within the EUnetHTA framework is a suitable starting point for the development of a joint HTA view on clinical evidence [19]. Nevertheless, specific topics and issues that are driving HTA decisions still differ considerably across the three countries. Those differences need to be addressed when further implementing the current European Commission proposal into HTA appraisal reality.
